# Mobile Health Coaching on Nutrition and Lifestyle Behaviors for Subfertile Couples Using the Smarter Pregnancy Program: Model-Based Cost-Effectiveness Analysis

**DOI:** 10.2196/13935

**Published:** 2019-10-23

**Authors:** Elsje C Oostingh, Robbin H Ophuis, Maria PH Koster, Suzanne Polinder, Hester F Lingsma, Joop SE Laven, Régine PM Steegers-Theunissen

**Affiliations:** 1 Erasmus University Medical Center Rotterdam Netherlands

**Keywords:** preconception, subfertility, IVF treatment, pregnancy, cost-effectiveness

## Abstract

**Background:**

The health care costs for reproductive care have substantially increased with the use of in vitro fertilization (IVF) treatment. The mobile health (mHealth) coaching program Smarter Pregnancy is an effective intervention to improve nutrition and lifestyle behaviors and pregnancy rates in (sub)fertile couples, including those who undergo IVF treatment. Therefore, we hypothesize that this mHealth program can also reduce health care costs associated with IVF treatment.

**Objective:**

This study aimed to evaluate the cost-effectiveness of the mHealth coaching program Smarter Pregnancy and compare it to usual care in women of subfertile couples who start their first IVF cycle.

**Methods:**

This model-based cost-effectiveness analysis was performed on data from couples undergoing IVF treatment at the Erasmus MC, University Medical Center Rotterdam. A decision tree model was used to assess the incremental cost-effectiveness ratio (ICER) of ongoing pregnancies and costs of use of the mHealth program as compared to usual care. A probabilistic sensitivity analysis was performed to consider the uncertainty surrounding the point estimates of the input parameters.

**Results:**

Based on our model including 793 subfertile women undergoing IVF treatment, use of the mHealth program resulted in 86 additional pregnancies and saved €270,000 compared to usual care after two IVF cycles, with an ICER of –€3050 (95% CI –3960 to –540) per additional pregnancy. The largest cost saving was caused by the avoided IVF treatment costs. Sensitivity analyses showed that the mHealth program needs to increase the ongoing pregnancy rate by at least 51% after two IVF cycles for cost saving.

**Conclusions:**

The mHealth coaching program Smarter Pregnancy is potentially cost saving for subfertile couples preceding their first IVF treatment. Implementation of this mHealth program in routine preconception care for subfertile couples should be seriously considered, given the relatively low costs and promising cost-effectiveness estimates.

## Introduction

Since the pioneer work of Edwards and Steptoe, in vitro fertilization (IVF) has become an indelible technology in modern era. Although the ongoing pregnancy rate after IVF treatment has tremendously increased [1], subfertility remains a worldwide problem affecting approximately 12% of couples of reproductive age [2]. In addition to the medical causes of subfertility, poor nutrition and lifestyle behaviors can impair fertility as well [3]. The mobile health (mHealth) coaching program Smarter Pregnancy [4,5] was developed to motivate (sub)fertile couples to adopt healthy nutrition and lifestyle behaviors. In a survey among (sub)fertile couples and a primary analysis of a randomized controlled trial (RCT) among couples with an IVF treatment indication, we showed that online coaching of participants resulted in significant improvements of their nutrition and lifestyle behaviors [6,7]. Moreover, our survey also showed that improvements in nutritional behavior lead to an increase in ongoing pregnancy rates in fertile and subfertile couples with and without IVF treatment [8]. The health care and societal costs of IVF treatment are substantial [9], and we believe that many costs can be saved when a healthy lifestyle is adopted. Here, we aim to assess the cost-effectiveness of the use of this mHealth program compared to usual care in subfertile women preceding their first IVF treatment.

## Methods

### Study Population

**Figure 1:**
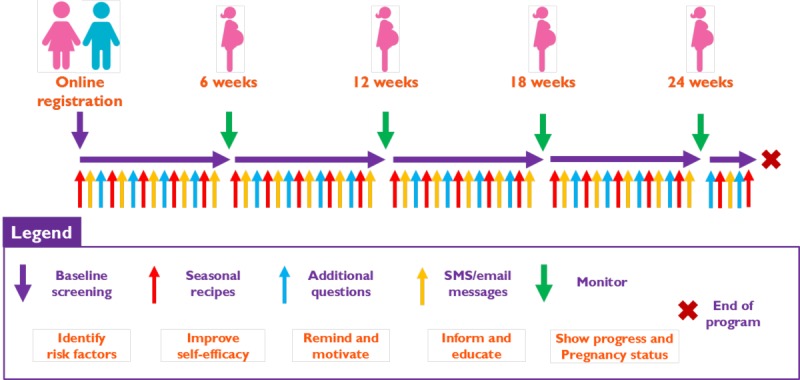
Overview of both the intervention and control groups during their enrollment in the Smarter Pregnancy randomized controlled trial. Adapted from van Dijk et al [<xref ref-type="bibr" rid="ref10">10</xref>]. SMS: short message service.

The data were derived from a modelled study population consisting of subfertile women undergoing their first IVF treatment at the Erasmus MC, University Medical Center Rotterdam, the Netherlands. The data of the RCT were used to model nutrition and lifestyle behaviors. In this RCT, participants were randomly assigned to the intervention or control group. Participants of the intervention group received the complete coaching program and were coached on a maximum of five nutrition and lifestyle behaviors: vegetable, fruit, and folic acid supplement intake, smoking, and alcohol consumption. Participants of the control group only received a diminished version of the program. At several time points, all participants were asked to fill out questionnaires about their nutrition and lifestyle behavior. In this way, change in behavior could be measured. Participants of the RCT started the program at a maximum of 2 months before start of their IVF treatment, and the program lasted for a period of 24 weeks (Figure 1). The study protocol and primary results of the RCT on the improvement of these behaviors have been published elsewhere [6,10]. In brief, participants in the intervention group showed a significantly larger improvement in inadequate behavior compared to the control group [6].

### Model

**Figure 2:**
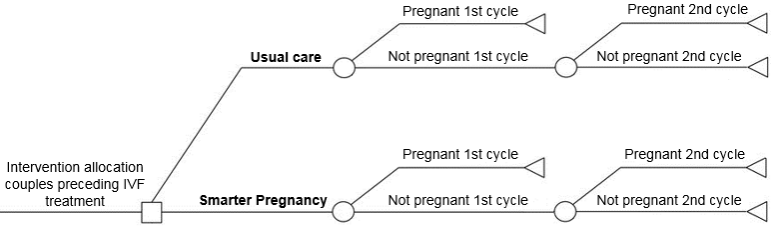
Decision tree model. IVF: in vitro fertilization.

A decision tree model was constructed using Microsoft Excel (version 2010; Microsoft Corporation, Redmond, Washington) to assess the incremental ongoing pregnancies following the first IVF cycle and the costs of the mHealth program as compared to usual care (Figure 2). Ongoing pregnancy was defined as a vital pregnancy at 12 weeks of gestation. Women of subfertile couples who underwent their first IVF treatment in 2015 entered the model (n=793). A second IVF cycle was started if the first cycle did not result in an ongoing pregnancy. Pregnancy outcome following the second IVF cycle was the endpoint of the model. This short-term evaluation should therefore be considered a first indication of cost-effectiveness of the mHealth program.

### Model Scenarios

The usual care scenario reflects usual IVF treatment in the Netherlands. We assumed that all women received an elective single embryo transfer and that pregnancy rates in usual care are 33% for the first IVF cycle and 23% for the second cycle [11]. We furthermore assumed that all women in the intervention scenario were offered the mHealth program (100% coverage). This program was not offered in the usual care scenario (0% coverage). The intervention adherence rate was set at 70%, based on RCT data in which 70% of participants in the intervention group completed the coaching [6].

### Model Parameters

Analyses were performed from a health care and societal perspective. The health care perspective includes costs related to the mHealth program [12], all costs associated with IVF treatment (eg, laboratory and hospital costs), and other relevant health care costs (eg, general practitioner visits). The societal perspective includes all health care costs plus costs outside the health care sector (eg, costs due to absence at work). The model parameters, including their distributions and sources, are reported in Table 1. Ongoing pregnancy rates after the first and second IVF cycle for the Smarter Pregnancy scenario and the usual care scenario were based on our previous study in the same setting [8] and others [13]. A detailed description of the cost calculations has been provided by Fiddelers et al [9]. All costs were expressed in euros (€) for the reference year 2016 based on the Dutch price index [14].

**Table 1:** Model input parameters.

Input parameter			Deterministic value	Probabilistic distribution	Source
**IVF^a^ costs (per cycle), €**					
	**Hospital costs**				
		Hormone stimulation - medication	1580	Fixed	Fiddelers et al [9]
		Hormone stimulation - hospital care	331	Fixed	Fiddelers et al [9]
		Ovum pick-up	596	Fixed	Fiddelers et al [9]
		Lab	1339	Fixed	Fiddelers et al [9]
		Embryo transfer	316	Fixed	Fiddelers et al [9]
		Other	295	Gamma	Fiddelers et al [9]
	**Other health care costs**				
		General practitioner	3	Gamma	Fiddelers et al [9]
		Other	13	Gamma	Fiddelers et al [9]
	**Costs outside health care^b^**				
		Sick leave	569	Gamma	Fiddelers et al [9]
		Leave of absence	141		Fiddelers et al [9]
		Loss of leisure time	73	Gamma	Fiddelers et al [9]
		Out of pocket expenditures	77	Gamma	Fiddelers et al [9]
		Informal care	32	Gamma	Fiddelers et al [9]
		Other	22	Gamma	Fiddelers et al [9]
**Intervention costs, €**					
		Smarter Pregnancy program costs	61^c^	Gamma	Luyendijk [12]
**Lifestyle costs^b^, €**					
		Folic acid supplement use	64	Fixed	Luyendijk [12]
		Healthy nutrition	113	Fixed	Luyendijk [12]
		Smoking	1,223	Fixed	Based on data from [15] and [16]
		Alcohol consumption	913	Fixed	Based on data from [17] and [18]
**Pregnancy rates usual care**					
		First IVF cycle	0.329	Beta	Based on Wade et al [11]
		Second IVF cycle	0.229	Beta	Based on Wade et al [11]
**Pregnancy rate intervention**					
		First IVF cycle - 65% increase	0.543	Beta	Based on Twigt et al [8]
		Second IVF cycle - 65% increase	0.443	Beta	Based on Twigt et al [8]

^a^IVF: in vitro fertilization.

^b^Only included in the analysis from a societal perspective. We assumed that the participants who smoke use 10 cigarettes per day (average daily use of smokers in the Netherlands) and that alcohol consumers drink one alcoholic beverage per day.

^c^Based on the annual tariff. This is considered to be an indication for the actual costs, which mainly consist of maintenance, insurance, overhead, and text messages.

### Cost-Effectiveness Analysis

The primary effect outcome measure was expressed as the number of ongoing pregnancies after two IVF cycles. Incremental cost-effectiveness ratios (ICERs) from health care and societal perspectives were calculated by dividing the difference in costs between the Smarter Pregnancy scenario and the usual care scenario by the difference in the number of ongoing pregnancies in both scenarios. The ICER represents the estimated costs of one additional ongoing pregnancy.

A probabilistic sensitivity analysis was performed to consider the uncertainty surrounding the point estimates of the model input parameters. Probabilistic distributions were assigned to the parameters (Table 1). Thereafter, 1000 model iterations were performed by drawing random values from the distributions assigned to the input parameters. We calculated the average costs and ongoing pregnancies by averaging these 1000 iterations. We performed deterministic sensitivity analyses to investigate the impact of changing several key parameters of the model: the coverage and adherence rate of the mHealth program and the chance of an ongoing pregnancy following the use of this program.

## Results

**Figure 3:**
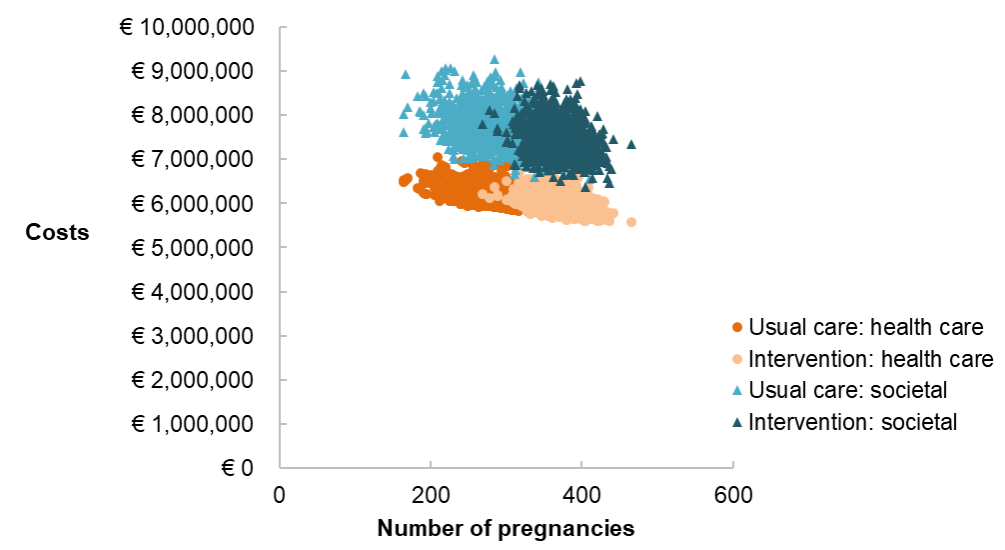
Costs and effects (ongoing pregnancy rate) of the mobile health coaching program Smarter Pregnancy (intervention) and usual care, categorized as per health care and societal perspectives.

**Figure 4:**
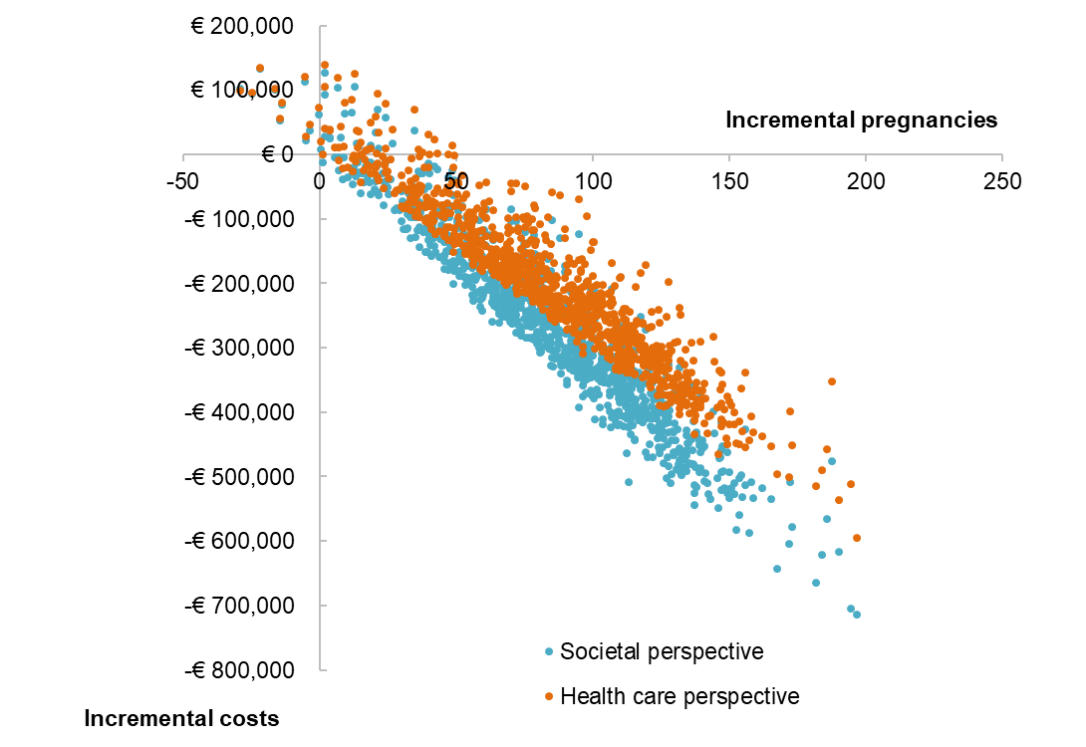
Incremental cost-effectiveness ratios generated by 1000 model simulations, categorized as per health care and societal perspectives.

Based on our model including 793 women, the mHealth scenario resulted in 369 pregnancies (47%; 95% CI 317-422) and the usual care scenario resulted in 283 pregnancies (36%; 95% CI 209-363) after two IVF cycles (Figure 1). The average health care costs for the mHealth and the usual care scenario were €6,008,500 (95% CI 5,671,000-6,505,000) and €6,214,800 (95% CI 5,839,500-6,730,300), respectively. The average societal costs for the mHealth and the usual care scenarios were €7,492,400 (95%CI 6,821,300;8,369,400) and €7,762,400 (95%CI 7,008,500-8,716,800), respectively (Figure 3). The ICERs from health care and societal perspectives per additional ongoing pregnancy equaled –€2250 (95%CI –3030 to –760) and –€3050 (95% CI –3960 to –540), respectively. Figure 4 shows that almost all ICERs are located in the southeast quadrant of the cost-effectiveness plane, indicating that the use of the mHealth program is cost saving.

The sensitivity analyses (Table 2) showed that the mHealth program is cost saving, on an average, but the uncertainty surrounding the ICERs increases when the intervention is less effective due to a lower compliance and ongoing pregnancy rate. For example, use of the mHealth program should increase the ongoing pregnancy rate by at least 51% for it to be cost saving compared to usual care when a 70% adherence rate is assumed. Otherwise, given an increased pregnancy rate of 65%, the compliance to Smarter Pregnancy should be at least 49% for it to remain cost saving.

**Table 2:** Results of the sensitivity analyses.

Result		Mean number of incremental pregnancies	Mean incremental societal costs, €	Mean ICER^a^ societal perspective (95%CI), €
Main analysis^b^		86	–270,000	–3050 (–3960 to –540)
**Sensitivity analyses**				
	85% intervention compliance	105	–340,200	–3210 (–3960 to –1630)
	55% intervention compliance	63	–192,000	–2840 (–3920 to –120)
	45% increase in pregnancy rate (0.477)	64	–186,300	–3070 (–5610 to 1620)
	25% increase in pregnancy rate (0.411)	40	–98,300	–2300 (–9610 to 9520)
	70% intervention coverage	62	–187,600	–2840 (–3930 to –540)
	85% intervention coverage	74	–227,400	–2850 (–3900 to –710)
	Worst-case scenario^c^	21	–37,300	–1270 (–20,900 to 13,200)
	Best-case scenario^d^	123	–408,900	–3600 (–3900 to –1850)

^a^ICER: incremental cost-effectiveness ratio.

^b^100% intervention coverage, 70% intervention compliance, 65% increase in pregnancy rate.

^c^70% intervention coverage, 55% intervention compliance, 25% increase in pregnancy rate.

^d^100% intervention coverage, 100% intervention compliance, 65% increase in pregnancy rate.

## Discussion

### Principal Findings

This model-based study, determining the estimates of available data, showed that the use of the mHealth program would result in 86 additional pregnancies and a reduction of €270,000 compared to usual care after two IVF cycles, resulting in an ICER of –€3050 per additional ongoing pregnancy. Sensitivity analyses showed that the use of this mHealth program is cost saving when the ongoing pregnancy rate increases to at least 51% after two cycles of single embryo-transfer IVF treatment.

### Strengths and Limitations

A strength of our model is the combined use of evidence-based data of the population, clinical effectiveness, compliance, and costs to support decision making. Although model parameters would ideally be based on meta-analyses or larger datasets, these were unavailable. Since the Smarter Pregnancy RCT is ongoing, assumptions regarding ongoing pregnancy rates had to be made based on our previous data. In economic evaluations, a time horizon that is long enough to capture all relevant costs and effects is preferred [19]. Our study was limited to two IVF cycles, which may be relatively short. However, as the endpoint of our study was to assess the incremental ongoing pregnancy rate, other costs and long-term reproductive and health outcomes were not considered.

We evaluated single embryo transfers only, because in the Netherlands, this is the most common IVF strategy. Therefore, costs and ongoing pregnancy rates of other IVF strategies will be different [13].

### Comparison With Prior Work

Several studies have investigated the effectiveness of nutrition and lifestyle interventions preceding fertility treatment. However, most of these studies focus on specific patient groups such as obese or anovulatory women [20,21]. In accordance with our findings, the study by Van Oers et al [22] showed that lifestyle intervention preceding fertility treatment was cost-effective in terms of achieving an ongoing pregnancy within 24 months.

The difference in average societal costs and health care costs was relatively small, indicating that the addition of the non-healthcare costs had no substantial impact on the ICER. Because nutrition and lifestyle interventions in preconception care have relatively low additional budget impact, we expect that the chance that the mHealth program is not cost-effective would be low [23].

### Conclusions

Our results show that the mHealth coaching program Smarter Pregnancy is potentially cost saving for subfertile couples preceding their first IVF treatment. Although our results are promising, our model requires further validation based on actual data on ongoing pregnancy rates from the Smarter Pregnancy RCT in order to establish the relative cost-effectiveness of the mHealth program with greater certainty. Implementation of this mHealth program in routine preconception care of subfertile couples should be seriously considered, given the relatively low intervention costs and promising cost-effectiveness estimates.

## Abbreviations

ICER: incremental cost-effectiveness ratio
IVF: in vitro fertilization
mHealth: mobile health
RCT: randomized controlled trial
